# Supplementary material for: Non-Curative Treatment Choices in Colorectal Cancer: Predictors and Between-Hospital Variations in Denmark: A Population-Based Register Study
Source: Cancers (Basel). 2024 Jan 15;16(2):366. doi: 10.3390/cancers16020366 (PMC10814909; doi:10.3390/cancers16020366)
Supplement: Supplementary file 1 [file cancers-16-00366-s001.zip › cancers-2805816-supplementary.pdf]

Supplementary Table S1. Overview of inclusion and exclusion criteria and changes over the study period regarding national recommendation of treatment with adjuvant oncological treatment after curative resection of stage II and III colorectal cancer. (+) indicates inclusion and (-) exclusion.

	**Colon cancer**										**Rectal cancer**									
	2009	2010	2011	2012	2013	2014	2015	2016	2017	2018	2009	2010	2011	2012	2013	2014	2015	2016	2017	2018
**UICC Stage II**																				
Low differentiation of tumor	+	+	+	+	+	+	+	+			+	+	+							
Signet ring cell carcinoma									+	+				+	+	+	+	+	+	+
pT4	+	+	+	+	+	+	+	+	+	+	+	+	+	+	+	+	+	+	+	+
Perineural or venous invasion	+	+	+	+	+	+	+	+			+	+	+							
< 12 lymph nodes examined in specimen	+	+	+	+	+	+	+	+	+	+	+	+	+	+	+	+	+	+	+	+
R1 resection or CRM ≤1mm											+	+	+	+	+	+	+	+	+	+
Anastomotic leakage grade B or C									+	+				+	+	+	+	+	+	+
Neo-adjuvant therapy																	-	-	-	-
dMMR				-	-	-	-	-	-	-				-	-	-	-	-	-	-
Performance status >2									-	-									-	-
> 75 years old									-	-									-	-
>80 years old	-	-	-	-	-	-	-	-	-	-	-	-	-	-	-	-	-	-	-	-
**UICC Stage III**	+	+	+	+	+	+	+	+	+	+			+	+	+	+	+	+	+	+
Low differentiation of tumor or mucinous											+	+								
pT4											+	+								
Perineural or venous invasion											+	+								
< 12 lymph nodes examined in specimen											+	+								
R1 resection or CRM ≤1mm											+	+								
Neo-adjuvant therapy																	-	-	-	-
>80 years old	-	-	-	-	-	-	-	-	-	-	-	-	-	-	-	-	-	-	-	-

Abbreviations: UICC, Union for International Cancer Control; CRM, Circumferential resection margin; dMMR, deficient Mismatch Repair status
